# Case report: Gastric cancer‐associated membranous nephropathy that recurred after complete remission and formation of peritoneal dissemination

**DOI:** 10.1002/ccr3.2002

**Published:** 2019-02-06

**Authors:** Nobuyuki Kajiwara, Noriko Wada, Takuya Kusumoto, Yusuke Akamaru, Hiroshi Ohashi, Kazuyuki Hayashi

**Affiliations:** ^1^ Department of Nephrology Ikeda City Hospital Ikeda Japan; ^2^ Department of Gastroenterological Surgery Ikeda City Hospital Ikeda Japan; ^3^ Post Graduate Clinical Education Center Ikeda City Hospital Ikeda Japan; ^4^ Department of Pathology Ikeda City Hospital Ikeda Japan

**Keywords:** complete remission, gastric cancer, membranous nephropathy, onco‐nephrology, paraneoplastic glomerulopathy, paraneoplastic syndrome, peritoneal dissemination, recurrence

## Abstract

Membranous nephropathy associated with malignant neoplasm may remit completely with treatment of the underlying disease. In such cases, recurrence is very rare. However, after a recurrence, attention should be paid to the possible recurrence of the underlying disease.

## 1 INTRODUCTION

Membranous nephropathy (MN) is sometimes associated with malignant neoplasm and may improve with treatment of the underlying disease. On the other hand, few reports have described MN recurrence/relapse associated with the recurrence of malignant neoplasm. We report herein the case of a patient who was found to have gastric cancer at the onset of nephrotic syndrome associated with MN, which remitted completely after surgery and chemotherapy, and who experienced recurrence of MN 7 years postoperatively and was diagnosed with peritoneal dissemination 4 years later.

## 2 CASE HISTORY

**Figure 1:**
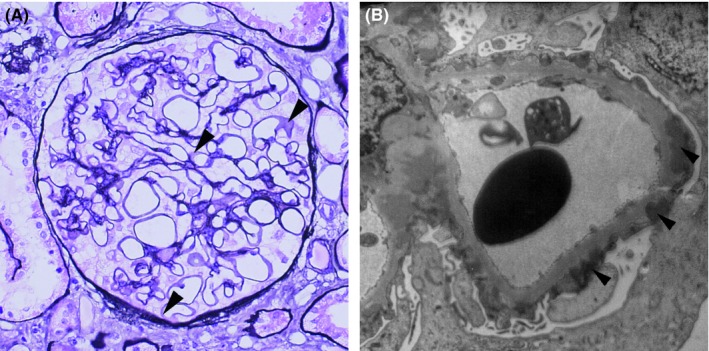
A, Periodic acid methenamine silver stain of the first renal biopsy specimen. Thickening of the glomerular basement membrane (GBM) was not apparent, but bubbly appearance was suspected in parts of the GBM (arrowheads). The fluorescent antibody technique detected no significant deposition of IgG, C3, or other immunoglobulins. B, Electron micrograph. Subepithelial presence of electron‐dense deposits against the GBM (arrowheads). Diagnosed as stage I‐II MN

**Figure 2:**
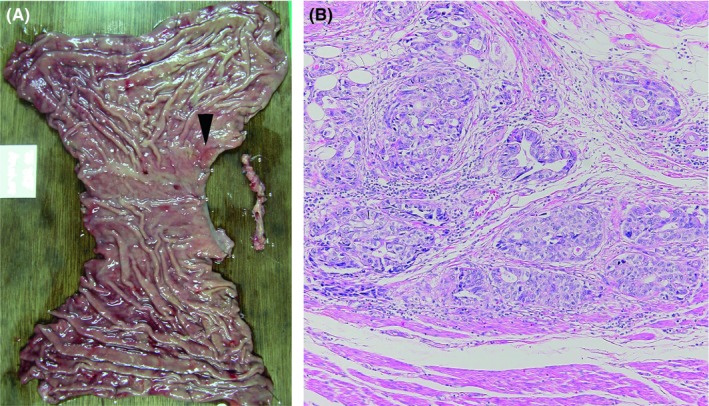
A, Resected specimen of the peripheral gastric cardia. Presence of 0‐IIa+IIc gastric cancer on the wall immediately anterior to the cardia (arrowhead). B, Tissue image. Presence of moderately differentiated adenocarcinoma centered around the submucosal layer with some slight muscle invasion

**Figure 3:**
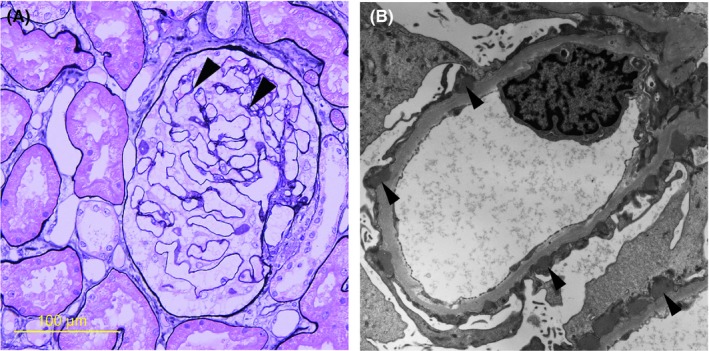
A, Periodic acid methenamine silver stain of the second renal biopsy specimen. Thickening of the glomerular basement membrane (GBM) was not apparent, but bubbly appearance was suspected in parts of the GBM (arrowheads). The fluorescent antibody technique detected no significant deposition of IgG, C3, or other immunoglobulins. B, Electron micrograph. Subepithelial presence of electron‐dense deposits against the GBM (arrowheads). Diagnosed as stage I MN recurrence

**Figure 4:**
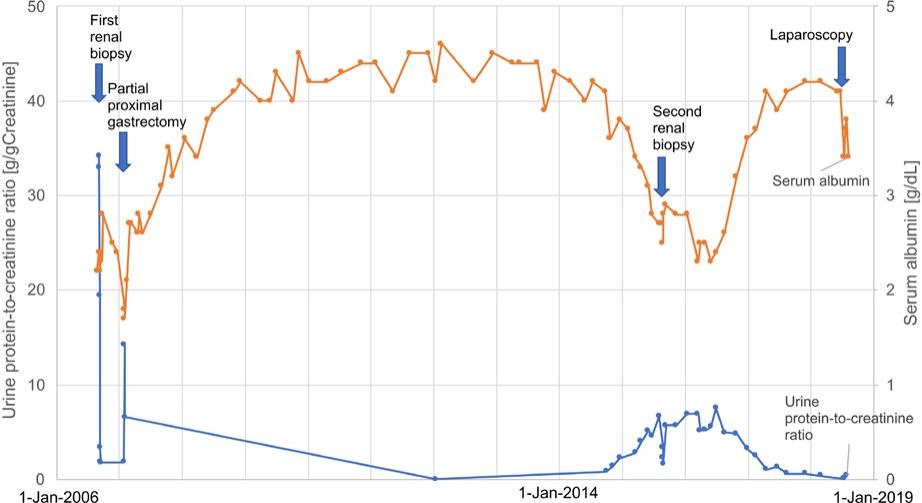
A graph showing changes in urine protein‐to‐creatinine ratio (blue line) and serum albumin value (orange line)

**Figure 5:**
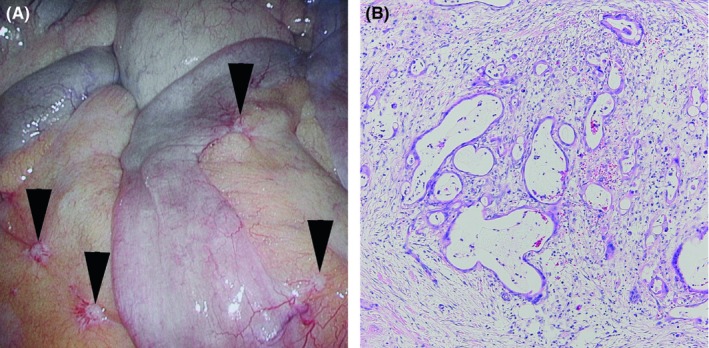
A, Macroscopic finding of the peritoneum through a laparoscope. Presence of multiple peritoneal white nodules (arrowheads). B, Tissue image. Image of well‐ to moderately differentiated adenocarcinoma

A 55‐year‐old Japanese male, whose 2005 health checkup revealed proteinuria for the first time, developed lower limb edema in August 2006 and was diagnosed with nephrotic syndrome. Renal biopsy in September yielded a diagnosis of MN (Figure 1A,B). Gastric cancer was identified and the patient underwent proximal gastrectomy and jejunal pouch interposition reconstruction in January 2007 (Figure 2A). The histologic diagnosis was T2N1M0 pStage IIA R0 tubular adenocarcinoma, moderate differentiated type (Figure 2B). (This pathological stage was based on Japanese Classification of Gastric Carcinoma—2nd English Edition).^1^ The patient received 12 months of postoperative adjuvant chemotherapy with tegafur/gimeracil/oteracil potassium (S‐1). Although he received no steroids or immunosuppressants to treat MN, proteinuria gradually decreased. In 2011, proteinuria remained negative according to the dipstick method, and the urine protein‐to‐creatinine ratio was 0.033 g/gCr, leading to the diagnosis of complete remission. The patient experienced proteinuria again in May 2014, developed nephrotic syndrome in June 2015, and was diagnosed with MN after a second renal biopsy in August (Figure 3A,B). Steroid or immunosuppressant treatment was again withheld, but a decreasing trend in proteinuria was noted (Figure 4). In May 2018, the patient complained of nausea and abdominal distension and contrast‐enhanced abdominal computed tomography and ^18^F‐fluoro‐2‐deoxy‐D‐glucose positron emission tomography showed findings suggestive of widespread peritoneal dissemination in the abdominal cavity. Esophago gastro duodenoscopy, total colon endoscopy, and contrast‐enhanced neck and chest computed tomography yielded no finding of another malignant neoplasm. Staging laparoscopy in July revealed widespread multiple peritoneal nodules (Figure 5A), biopsy confirmed well‐ to moderately differentiated adenocarcinoma (Figure 5B), and no findings indicated primary malignant neoplasms in other organs, so the patient was diagnosed with peritoneal dissemination of gastric cancer and began chemotherapy with S‐1 and oxaliplatin.

## 3 DISCUSSION

In adult patients, "complete remission" of nephrotic syndrome is "24‐hour protein excretion is <0.3 g" by definition.^2^ The present case was diagnosed as secondary MN at the initial onset because complete remission was seen with treatment for gastric cancer. The recurrence was at first thought to represent primary MN, given the lack of apparent signs of gastric cancer recurrence or metastasis. However, secondary MN was diagnosed after peritoneal dissemination was confirmed 4 years later. Nevertheless, proteinuria was milder at diagnosis of peritoneal dissemination than at MN recurrence, showing an unexplained absence of a corresponding progression in MN as the primary disease worsened.

Membranous nephropathy associated with malignant neoplasm is considered a paraneoplastic syndrome (PNS) or paraneoplastic glomerulopathy. PNS has been defined as follows^3^, ^4^, ^5^: “The term paraneoplastic syndrome refers to clinical manifestations that are not directly related to tumor burden, invasion, or metastasis, but are caused by the secretion of tumor cell products such as hormones, growth factors, cytokines, and tumor antigens.” According to Ronco, the diagnosis of PNS theoretically relies on three strong criteria^5^: “First, a clinical and histologic remission occurs after complete surgical removal of the tumor or chemotherapy‐induced complete remission of the disease. Second, a renal relapse accompanies recurrence of the neoplasia. Third, a pathophysiologic link is established between the two diseases, including the detection of tumor antigens and antitumor antibodies within subepithelial immune deposits.” Clinically, however, few reports have described neoplastic recurrence‐associated recurrence/relapse of MN. Leeaphorn et al^6^ reported that MN is often associated with cancer of the lungs, prostate, and GI system. A search of PubMed using a query consisting of “membranous nephropathy,” and “lung cancer,” “esophageal cancer,” “gastric cancer,” “colon cancer,” “rectal cancer,” “prostate cancer,” or “breast cancer” returned no case reports of MN and cancer recurrences after complete remission of MN was achieved with treatment of one of the seven types of cancer. One case report described glomerulopathy that improved with treatment for metastatic gastric cancer lesions, but relapsed in association with the worsening of other metastatic lesions. A report by Robinson et al described a patient with nephrotic syndrome due to membranoproliferative glomerulonephritis associated with vertebral metastasis of adenocarcinoma of unknown primary who achieved incomplete remission with radiotherapy, but suffered a recurrence of nephrotic syndrome associated with metastases to the pelvis and brain and in whom gastric cancer was later found.^7^ The report indicated that proteinuria decreased to <0.4 g/d after radiotherapy, but was not in complete remission. Three reports described cases of lung cancer‐associated MN that entered remission with treatment for lung cancer, but was exacerbated by recurrence or metastasis of the lung cancer. Boon et al reported a case of nephrotic syndrome due to MN associated with small‐cell lung cancer, in which proteinuria decreased to 1 g/d after treatment for lung cancer, but increased to 1.7 g/d following brain metastasis.^8^ Yangui et al reported a case of nephrotic syndrome due to MN associated with small‐cell lung cancer, in which proteinuria decreased to 1.5 g/d after treatment for lung cancer, but, after the lung cancer recurred, renal function decreased rapidly, and nephrotic syndrome also recurred.^9^ Crawford et al reported the case of a patient with MN associated with nonsmall‐cell lung cancer who achieved remission after chemoradiation therapy for lung cancer, but whose proteinuria increased along with the recurrence of lung cancer.^10^ In all three cases, following treatment for lung cancer, proteinuria decreased, but was not less than 0.3 g/d, showing no complete remission. We believe the absence of reports of MN recurring after complete remission following treatment for malignant neoplasm is attributable to the fact that malignant neoplasm almost never recurs if malignant cells are successfully removed to the extent that complete remission of MN is achieved.

Membranous nephropathy is a rare disease (annual incidence of 2.8 per 100 000 over the age of 60) compared with malignancy (annual incidence over 1000 per 100 000 in the same age period).^5^ Then, the true incidence of glomerular disease associated with malignancy is not known.^3^ On the other hand, the estimated prevalences of malignancy in patient with MN are 10.0% in Europe and the USA,^6^ 3.1% in China,^11^ and 1.0% in Japan.^12^

In most cases of MN considered to represent PNS, the corresponding neoplasm would develop within ±12 months.^13^, ^14^ In our case, however, peritoneal dissemination occurred approximately 4 years after MN recurrence. We speculate that even in cases of PNS, the two events may be separated by a long interval.

Primary MN has been said to be predominantly associated with deposition of immunoglobulin (Ig)G4, and secondary MN, with deposition of IgG1 and IgG2.^15^ In the present case, the fluorescent antibody technique failed to reveal any apparent deposition of IgG or to identify the IgG subclass. We have seen in our hospital 2 patients with MN diagnosed by electron microscopy with the fluorescent antibody technique failing to detect IgG deposition: One was associated with bucillamine, and the other, with prostate cancer. Due to technical issues, the fluorescent antibody technique may have weak power for detecting IgG deposition in secondary MN. The presence of anti‐PLA2R antibodies, which is considered rare in secondary MN,^4^ was not investigated in the present case.

Gastric cancer reportedly recurred postoperatively within 5 years in 66%‐77% of patients with early cancer and in 91.9% of patients with advanced cancer.^16^, ^17^ Patients with gastric cancer rarely develop peritoneal dissemination 11 years postoperatively, as in the present case. We believe the great reduction in cancer cell burden achieved with surgery and chemotherapy was the reason behind the temporary complete remission of MN and the long interval between the resection of the gastric cancer and the onset of peritoneal dissemination.

We have reported a rare but theoretically possible case of MN that went into complete remission after treatment for malignant neoplasm, but recurred along with the malignant neoplasm. This case shall serve as a reference for future discussions in onco‐nephrology.

## CONFLICT OF INTEREST

The authors have no conflict of interest to declare with regard to this report.

Consent: While ensuring the anonymity of the patient, we obtained the patient's written consent to report his case.

## AUTHOR CONTRIBUTION

NK: treated the renal lesions throughout their entire course and drafted the manuscript. NW and YA: treated the peritoneal dissemination. TK and HO: performed pathological examinations. KH: participated in the treatment of renal lesions. All authors contributed to the manuscript preparation and revisions and consented to be held accountable for the present report.

## References

[1] Japanese Gastric Cancer Association . Japanese classification of gastric carcinoma–2nd English edition. Gastric Cancer. 1998;1(1):10‐24. 11957040 10.1007/s101209800016

[2] Schrier RW . Diseases of the Kidney & Urinary Tract (8th ed.). Philadelphia: Lippincott Williams & Wilkins 2007;1613.

[3] Bacchetta J , Juillard L , Cochat P , Droz J‐P . Paraneoplastic glomerular diseases and malignancies. Crit Rev Oncol Hematol. 2009;70(1):39‐58. 18790651 10.1016/j.critrevonc.2008.08.003

[4] Cambier J‐F , Ronco P . Onco‐nephrology: glomerular diseases with cancer. Clin J Am Soc Nephrol. 2012;7(10):1701‐1712. 22904123 10.2215/CJN.03770412

[5] Ronco PM . Paraneoplastic glomerulopathies: new insights into an old entity. Kidney Int. 1999;56(1):355‐377. 10411717 10.1046/j.1523-1755.1999.00548.x

[6] Leeaphorn N , Kue‐A‐Pai P , Thamcharoen N , Ungprasert P , Stokes MB , Knight EL . Prevalence of cancer in membranous nephropathy: a systematic review and meta‐analysis of observational studies. Am J Nephrol. 2014;40(1):29‐35. 24993974 10.1159/000364782

[7] Robinson WL , Mitas JA , Haerr RW , Cohen IM . Remission and exacerbation of tumor‐related nephrotic syndrome with treatment of the neoplasm. Cancer. 1984;54(6):1082‐1084. 6467133 10.1002/1097-0142(19840915)54:6<1082::aid-cncr2820540625>3.0.co;2-3

[8] Boon E , Vrij A , Nieuwhof C , Van Noord J , Zeppenfeldt E . Small cell lung cancer with paraneoplastic nephrotic syndrome. Eur Respir J. 1994;7(6):1192‐1193. 7925893

[9] Yangui I , Msaad S , Smaoui M , et al. Small‐cell lung cancer and rapidly fatal nephritic syndrome. Rev Pneumol Clin. 2007;63(5 Pt 1):331‐334 [French]. 18166938 10.1016/s0761-8417(07)74212-8

[10] Crawford AR , Dworkin L , Leonard K , Khurshid H , Hepel JT . Recurrence of paraneoplastic membranous glomerulonephritis following chemoradiation in a man with non‐small‐cell lung carcinoma. Rare Tumors. 2013;5(2):62‐64. 23888216 10.4081/rt.2013.e16 PMC3719111

[11] Zeng C‐H , Chen H‐m , Wang R‐S , et al. Etiology and clinical characteristics of membranous nephropathy in Chinese patients. Am J Kidney Dis. 2008;52(4):691‐698. 18805348 10.1053/j.ajkd.2008.06.006

[12] Yokoyama H , Taguchi T , Sugiyama H , Sato H . Membranous nephropathy in Japan: analysis of the Japan Renal Biopsy Registry (J‐RBR). Clin Exp Nephrol. 2012;16(4):557‐563. 22358611 10.1007/s10157-012-0593-7

[13] Eagen JW . Glomerulopathies of neoplasia. Kidney Int. 1977;11(5):297‐303. 197291 10.1038/ki.1977.47

[14] Lien Y‐HH , Pathogenesis L‐W . diagnosis and management of paraneoplastic glomerulonephritis. Nat Rev Nephrol. 2011;7(2):85‐95. 21151207 10.1038/nrneph.2010.171 PMC3058941

[15] Ohtani H , Wakui H , Komatsuda A , et al. Distribution of glomerular IgG subclass deposits in malignancy‐associated membranous nephropathy. Nephrol Dial Transplant. 2004;19(3):574‐579. 14767011 10.1093/ndt/gfg616

[16] Sano T , Sasako M , Kinoshita T , Maruyama, . K. Recurrence of early gastric cancer. Follow‐up of 1475 patients and review of the Japanese literature. Cancer. 1993;72(11):3174‐3178. 8242540 10.1002/1097-0142(19931201)72:11<3174::aid-cncr2820721107>3.0.co;2-h

[17] Katai H , Maruyama K , Sasako M , et al. Mode of recurrence after gastric cancer surgery. Dig Surg. 1994;11(2):99‐103.

